# Analysis of Aspirin Use and Cardiovascular Events and Mortality Among Adults With Hypertension and Controlled Systolic Blood Pressure

**DOI:** 10.1001/jamanetworkopen.2022.6952

**Published:** 2022-04-12

**Authors:** Rita Del Pinto, Davide Pietropaoli, Giovambattista Desideri, Claudio Ferri

**Affiliations:** 1 Department of Clinical Medicine, Public Health, Life and Environmental Sciences, University of L'Aquila, L’Aquila, Italy; 2 Division of Internal Medicine and Nephrology, Center for Hypertension and Cardiovascular Prevention, San Salvatore Hospital, L’Aquila, Italy; 3 Geriatrics Unit, SS Filippo e Nicola Hospital, Avezzano, Italy

## Abstract

This cohort study investigates the association of aspirin use with risk of cardiovascular events among adults with hypertension and controlled systolic blood pressure.

## Introduction

Aspirin use for the primary prevention of atherosclerotic cardiovascular disease (ASCVD) has been questioned for having limited benefit in individuals with diabetes,^1^ neutral outcomes in individuals at low risk,^2^ and increased bleeding risk and mortality in healthy older individuals.^3,4^ Given the lack of specific updated evidence, we aimed to investigate whether aspirin use alongside guideline-directed blood pressure (BP) management targeting a systolic BP of less than 140 mm Hg is associated with decreased risk of a first cardiovascular event and improved survival among individuals with hypertension at increased risk of ASCVD.

## Methods

This cohort study report follows the Strengthening the Reporting of Observational Studies in Epidemiology (STROBE) reporting guideline for cohort studies. Given that this was a post hoc analysis of available data, the Institutional Review Board at the University of L’Aquila determined that this study did not require informed consent or local ethical review.

The Systolic Blood Pressure Intervention Trial (SPRINT)^5^ was a multicenter, treat-to-target, 2-arm randomized clinical trial conducted from 2010 to 2013 (median follow-up, 3.26 years) comparing intensive (<120 mm Hg) and standard (<140 mm Hg) guideline-directed BP-lowering strategies among patients with hypertension and no history of diabetes or stroke at increased risk of ASCVD. Alongside smoking cessation and dyslipidemia treatment, aspirin therapy was part of the background therapy recommendations.

We derived a primary prevention cohort by excluding participants with baseline cardiovascular disease (CVD). Individuals with chronic kidney disease were excluded owing to increased ischemic and hemorrhagic risk. Participants were included if valid baseline and consistent in-trial information on aspirin use (ie, the exposure) was available (eFigure in the Supplement).

All analyses were conducted using R statistical software version 4.0.2 (R Project for Statistical Computing). Data were analyzed from October 2021 to February 2022. Unpaired *t* and χ^2^ tests were used to test for differences in quantitative (ie, mean [SD]) and qualitative (ie, No. [%]) data, respectively. Statistical significance was set at *P* < .05, and *P* values were 2-sided. Missing data were not imputed. Patients who were exposed vs not exposed were 1:1 propensity-matched for randomization group, sex, age category, Black race, and having ever smoked. Race and ethnicity were self-reported in SPRINT to characterize the final study population; options included Black, Asian, Hispanic, White, and other. Black race was a prespecified subgroup of interest in SPRINT.

Risk of cardiovascular events (ie, the primary outcome, including adjudicated myocardial infarction, non–myocardial infarction acute coronary syndrome, stroke, acute heart failure, and CVD death) and all-cause mortality based on the exposure (expressed as hazard ratios with 95% CIs) were progressively adjusted for residual, noncollinear confounders (ie, age at randomization, current smoking status, serum creatinine level, and triglyceride level). Subgroup analyses (ie, by race and ethnicity, age category, current vs former smoking status, body mass index [BMI; calculated as weight in kilograms divided by height in meters squared], and statin level) were performed, and outcomes rates were assessed by intensity of systolic BP lowering. A sensitivity analysis was performed by censoring early primary and fatal events (occurring <1 and <2 years after enrollment, respectively). Additional information is in eMethods in the Supplement.

## Results

In total, 2664 participants in SPRINT (1332 participants/study group; 390 [29.3%] women; 326 individuals aged ≥75 y [24.5%]) were analyzed (Table). Individuals in the exposed group were older, had increased BMI and better lipid profiles, and were more likely to have never smoked and be taking a statin. No between-group differences in history of peptic ulcer, chronic liver disease, or daily nonsteroidal anti-inflammatory drug use were recorded.

**Table.** Demographic and Clinical Characteristics

Characteristic	Participants, No. (%) (N = 2664)^a^		*P* value	Missing values, No. (%)
	Exposed (n = 1332)	Not exposed (n = 1332)		
Women	390 (29.3)	390 (29.3)	>.99	0
Age, mean (SD)	67.7 (8.4)	65.9 (9.6)	<.001	0
Ages ≥75 y	326 (24.5)	326 (24.5)	>.99	0
Black race^b^	337 (25.3)	358 (26.9)	.38	0
Overall race and ethnicity^c^				
Black	337 (25.3)	358 (26.9)	<.001	0
Hispanic	80 (6.0)	224 (16.8)		0
White	895 (67.2)	717 (53.8)		0
Other	20 (1.5)	33 (2.5)		0
Intensive treatment group	665 (49.9)	665 (49.9)	>.99	0
Never smoked	625 (46.9)	616 (46.2)	.82	0
FRS, mean (SD)	17.1 (2.2)	17.2 (2.4)	.42	0
Creatinine, mean (SD), mg/dL	0.95 (0.17)	0.92 (0.17)	<.001	53 (0.2)
History of peptic ulcer	115 (8.6)	129 (9.7)	.38	0
Chronic hepatitis	21 (1.6)	35 (2.6)	.08	0
NSAID use	216 (16.2)	183 (13.7)	.08	0
Total cholesterol, mean (SD), mg/dL	188.2 (39.1)	197.2 (39.2)	<.001	0
Serum glucose, mean (SD), mg/dL	99.5 (14.5)	98.7 (13.2)	.14	0
HDL cholesterol, mean (SD), mg/dL	53.1 (15.1)	52.9 (14.4)	.75	0
Triglycerides, mean (SD), mg/dL	123.7 (84.7)	131.6 (112.1)	.04	0
BMI, mean (SD)	30.3 (5.4)	29.5 (5.3)	<.001	133 (0.5)
Statin use	665 (50.3)	358 (27.0)	<.001	159 (0.6)

Abbreviations: BMI, body mass index (calculated as weight in kilograms divided by height in meters squared); FRS, Framingham 10-year cardiovascular diseases risk score; HDL, high-density lipoprotein; NSAID, nonsteroidal anti-inflammatory drug.

SI conversion factors: To convert creatinine to micromoles per liter, multiply by 88.4; HDL and total cholesterol to millimoles per liter, multiply by 0.0259; serum glucose to millimoles per liter, multiply by 0.0555; triglycerides to millimoles per liter, multiply by 0.0113.

^a^ Exposed and nonexposed groups were propensity matched for treatment group, sex, age category, Black race, and having ever smoked.

^b^ Black race listed separately as a prespecified subgroup of interest in the Systolic Blood Pressure Intervention Trial that was used for propensity score matching.

^c^ Race and ethnicity were self-reported. Other race and ethnicity includes Asian and other racial and ethnic minority groups, including Indian, Hawaiian, and other unspecified racial and ethnic groups (eMethods in the Supplement).

Nonexposure was associated with decreased risk of the primary outcome (Figure, A), with consistent findings in subgroups of younger individuals, former and current smokers, and those on a statin (Figure, B). Similar results were found in the sensitivity analysis among 2444 participants. The primary outcome rates of exposed and nonexposed groups were similar independent of randomization group (standard: 5.85%; 95% CI, 4.24%-7.98% vs 3.60%; 95% CI, 2.37%-5.39%; *P* = .07; intensive: 4.66%; 95% CI, 3.24%-6.63% vs 2.56%; 95% CI, 1.54%-4.15%; *P* = .06).

**Figure.**
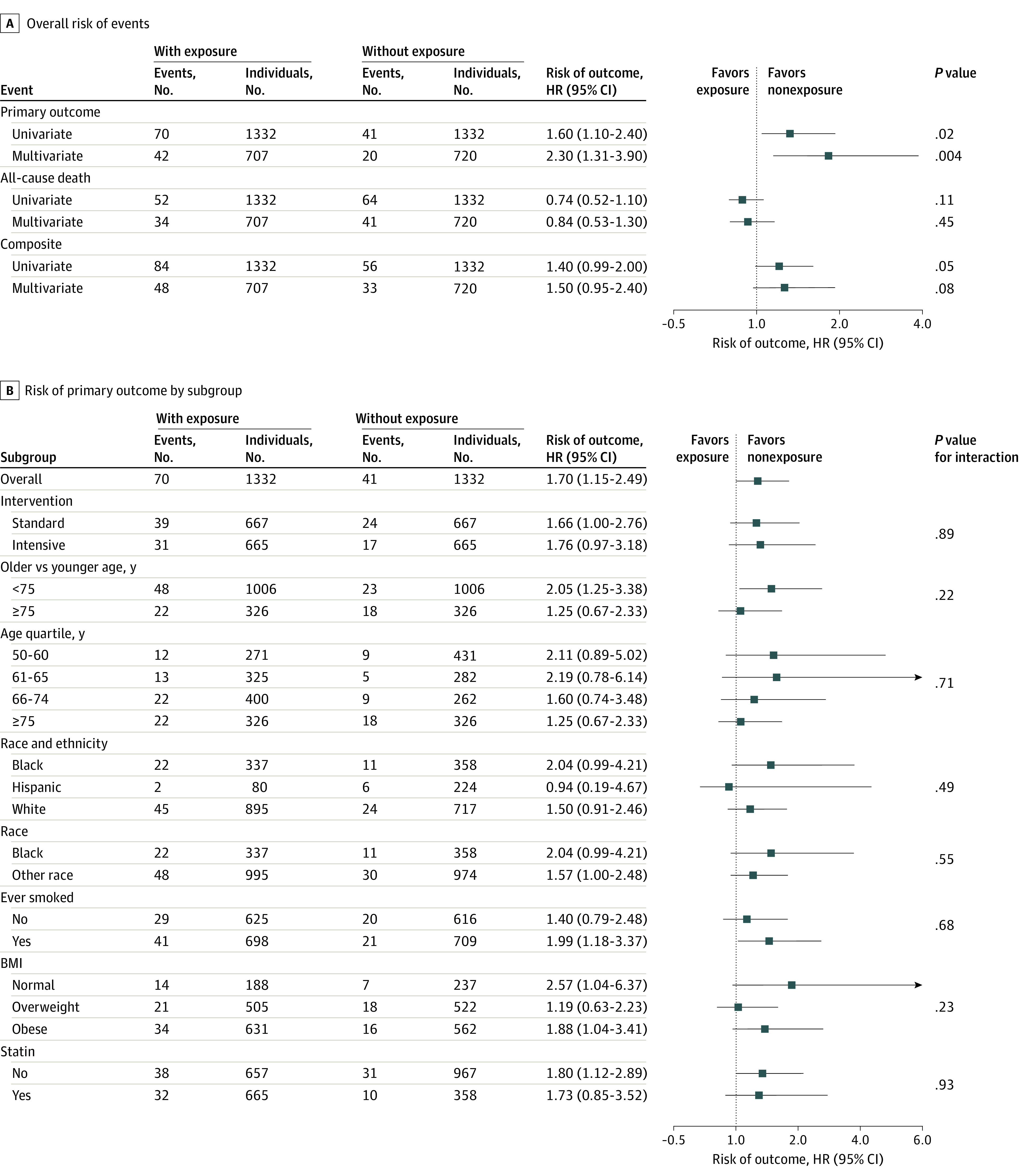
Risk of Cardiovascular Events and Death by Exposure The number of events per number of individuals was reported for exposed and nonexposed groups. *P* values were adjusted for multiple subgroups tested. Race and ethnicity were self-reported. See eMethods in the Supplement for details.

## Discussion

This cohort study has several limitations, including being a post hoc analysis of a trial not designed to study the association of aspirin with cardiovascular events, having a short follow-up time, and lacking data on aspirin initiation and bleeding events. However, our results suggest that the modern management of hypertension may have redefined the benefit associated with aspirin of decreased risk of a first cardiovascular event and improved survival in an experimental cohort of patients with hypertension and without diabetes, ASCVD, or chronic kidney disease at increased risk of ASCVD. The use of multiple antihypertensive drugs, the downward redefinition of BP targets, and the improvement of additional cardiovascular prevention strategies may have been paramount in the association with decreased ASCVD risk in the examined context.^6^ Long-term data on aspirin use in combination with emerging therapies for cardiovascular prevention may clarify the future role of this pivotal drug in similar clinical settings.

## Notes

Supplement.
eMethods.

eReferences.
**eFigure.** Data-Reduction Diagram
