# Supplementary material for: Analysis of Aspirin Use and Cardiovascular Events and Mortality Among Adults With Hypertension and Controlled Systolic Blood Pressure
Source: JAMA Netw Open. 2022 Apr 12;5(4):e226952. doi: 10.1001/jamanetworkopen.2022.6952 (PMC9006111; doi:10.1001/jamanetworkopen.2022.6952)
Supplement: Supplement. — eMethods. eReferences. eFigure. Data-Reduction Diagram [file jamanetwopen-e226952-s001.pdf]

Supplemental Online Content

Del Pinto R, Pietropaoli D, Desideri G, Ferri C. Analysis of aspirin use and cardiovascular events and mortality among adults with hypertension and controlled systolic blood pressure. *JAMA Netw Open.* 2022;5(4):e226952. doi:10.1001/jamanetworkopen.2022.6952

eMethods.
eReferences.
eFigure. Data-Reduction Diagram

This supplemental material has been provided by the authors to give readers additional information about their work.

© 2022 Del Pinto R et al. *JAMA Network Open.*

eMethods.

Study design and population

SPRINT was a two-arm, multicenter, randomized clinical trial conducted from 2010 to 2013 (median follow-up: 3.26 years) designed to test whether a systolic BP less than 120 mmHg compared with less than 140 mmHg reduces cardiovascular events and mortality in hypertensive patients at high cardiovascular risk, defined by the presence of at least one among: clinical or subclinical CVD other than stroke; CKD (estimated glomerular filtration rate [eGFR] 20-59 ml/min/1.73m^2); age ≥75 years; or 10-year Framingham Risk Score for CVD ≥15% [1,2]. Clinical CVD was defined as previous myocardial infarction (MI) with or without invasive treatment; carotid atherosclerosis with endarterectomy or stenting; peripheral artery disease with revascularization; acute coronary syndrome (ACS), or positive cardiac imaging study; significant stenosis (≥50% diameter) of a coronary, carotid, or lower extremity artery; and treated/untreated abdominal aortic aneurysm ≥5 cm [1]. Subclinical CVD included CAC score ≥ 400 Agatston units, ABI ≤0.90, and documented left ventricular hypertrophy [1].

Individuals with diabetes and/or with prior stroke were excluded from participation in the trial, and so were those with cardiovascular events or procedures as well as with hospitalization for unstable angina within the 3 months before enrollment.

Together with smoking cessation counseling and treatment of dyslipidemia, antithrombotic therapy with aspirin in SPRINT was part of the background therapy recommendations, and the decision to recommend daily aspirin and the dose was deferred to the participant's primary healthcare provider. Information on aspirin use was collected at baseline and annually thereafter based on a questionnaire.

The present study is a post hoc, exploratory analysis of aspirin use (exposure) in a primary prevention cohort derived from SPRINT by excluding participants with baseline clinical/subclinical CVD. Pre-existing CKD was also an exclusion criterion for its association with both ischaemic and hemorrhagic abnormalities. Participants were included if valid information on exposure (yes, no) was reported at baseline and if it was consistently confirmed thereafter until the closeout visit (**eFigure 1**). Analyses were performed from October 2021 to February 2022 following the Strengthening the Reporting of Observational Studies in Epidemiology (STROBE) reporting guideline for cohort studies [3].

Ethical considerations

Written informed consent for participation in the trial was collected by SPRINT investigators. As an analysis of existing and de-identified data, this study was deemed exempt from review by the Institutional Review Board at the University of L'Aquila.

Study outcomes

In SPRINT, the primary outcome was a composite of fatal/nonfatal cardiovascular events, i.e. adjudicated MI, ACS not resulting in MI (non-MI ACS), stroke, acute decompensated heart failure (HF), or death from CVD; or each single component [2]. The secondary outcome included all-cause mortality and its combination with the primary outcome [2]. In this post hoc analysis, the outcomes of interest were the occurrence of any fatal/nonfatal cardiovascular event, all-cause mortality, and their composite.

Statistical analysis

All analyses were conducted using R (v.4.0.2). After applying the predefined inclusion and exclusion criteria, we derived a primary prevention cohort of exposed (n. 1409) and nonexposed (n. 2190) individuals who reported persistence in aspirin use and nonuse, respectively, from the baseline visit onwards. Differences in clinical and demographic characteristics were evaluated with unpaired t-test for continuous variables (mean±standard deviation [SD]) and x^2-tests for categorical variables (N, %). Statistical significance was set at $p<0.05$ (two-sided). To exclude an exposure selection bias and sample discrepancy, a propensity score-matched (PSM) cohort was calculated using logistic regression analysis with 1:1 matching without replacement [4]. In order to avoid selection bias [5,6], the inclusion of covariates in the PSM model was restricted to those that impact on the primary outcome, namely randomization arm (intensive, standard), sex (male, female), age category (< 75 years, ≥75 years), Black race (yes, no), and having ever smoked (yes, no). Groups were exactly matched for randomization arm, sex, and age category (P value >.99), while they were nearest matched for Black race and smoking habits. The addition of further variables did not produce matched results. The PSM cohort consisted of n. 2664 individuals, equally distributed between exposed and nonexposed participants (n. 1332/group). Data were analyzed as recorded, without imputation for missing data.

We then determined the association of the exposure with outcomes in terms of hazard ratios (HR; 95% confidence intervals, CI) using Cox proportional hazard models progressively adjusted for age at randomization (univariable model), current smoking, creatinine, and triglycerides (multivariable model). The covariates included in the models met the assumption of proportionality. Collinearity among covariates was formally tested using the bootstrap stepwise algorithm. Subgroup analyses for crude HR for the primary outcome based on the exposure were performed by randomization arm, senior age category (< 75 years, ≥75 years), age quartiles (50-

60, 61-65, 66-74, and ≥75 years), statin use, BMI category (normal, overweight, obese), and smoking habits (former/current smokers; never smokers). Interactions between exposure and prespecified subgroups were assessed with a likelihood-ratio test for the interaction with the use of Hommel-adjusted P values [7]. A sensitivity analysis was also made where primary events that occurred before the first year and fatal events for any cause occurring before the second year after enrollment were censored [8,9], thus only including events attributable to in-trial conditions, namely intensive or standard BP control. Outcomes rates were evaluated within each treatment arm according to exposure, and the likelihood estimate for the difference between exposed/unexposed was evaluated using Yates correction for continuity.
Race/ethnicity was self-reported in SPRINT; options were defined in the trial protocol to characterize the final study population and included Black, Asian, Hispanic, White, Other (including minorities). In SPRINT, participants of Spanish, Hispanic, or Latino origin could select among being Rican (Puerto Rican), Cuban (Cuban), Mexican (Mexican, Mexican American, or Chicano), or other (unspecified); participants could self-define their race or ethnicity as White (White, Caucasian), Black (Black, African American), Indian (American Indian or Alaska Native), Hawaiian (Native Hawaiian or Pacific Islander), Asian (Asian), and other (unspecified). These data were then stratified before being accessible to the authors of this study in the 4 strata reported.

© 2022 Del Pinto R et al. *JAMA Network Open.*

eReferences.

1. Ambrosius WT, Sink KM, Foy CG, et al. The design and rationale of a multicenter clinical trial comparing two strategies for control of systolic blood pressure: the Systolic Blood Pressure Intervention Trial (SPRINT). *Clin Trials*. 2014;11(5):532-546.

2. SPRINT Research Group, Wright JT Jr, Williamson JD, et al. A Randomized Trial of Intensive versus Standard Blood-Pressure Control. *N Engl J Med*. 2015;373(22):2103-2116.

3. von Elm E, Altman DG, Egger M, et al. The Strengthening the Reporting of Observational Studies in Epidemiology (STROBE) statement: guidelines for reporting observational studies. *Ann Intern Med*. 2007;147(8):573-577.

4. D'Agostino RB Jr. Propensity score methods for bias reduction in the comparison of a treatment to a non-randomized control group. *Stat Med*. 1998;17(19):2265-2281.

5. Bergstra SA, Sepriano A, Ramiro S, Landewé R. Three handy tips and a practical guide to improve your propensity score models. *RMD Open*. 2019;5(1):e000953.

6. Brookhart MA, Schneeweiss S, Rothman KJ, Glynn RJ, Avorn J, Stürmer T. Variable selection for propensity score models. *Am J Epidemiol*. 2006;163(12):1149-1156.

7. G. H. A stagewise rejective multiple test procedure based on a modified Bonferroni test. *Biometrika*. 1988;75:383-386.

8. Chang TI, Reboussin DM, Chertow GM, et al. Visit-to-Visit Office Blood Pressure Variability and Cardiovascular Outcomes in SPRINT (Systolic Blood Pressure Intervention Trial). *Hypertension*. 2017;70(4):751-758.

9. Del Pinto R, Pietropaoli D, Dobre M, Ferri C. Prognostic importance of long-term SBP variability in high-risk hypertension. *J Hypertens*. 2020;38(11):2237-2244.

© 2022 Del Pinto R et al. *JAMA Network Open.*

eFigure. Data-Reduction Diagram

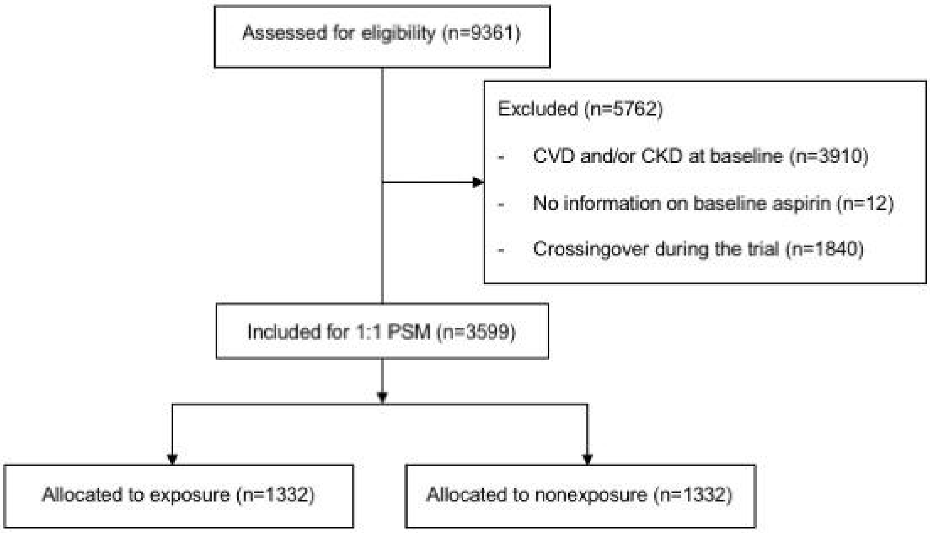

© 2022 Del Pinto R et al. *JAMA Network Open.*
